# Pregnancy outcomes in Takayasu arteritis patients: a systematic review and meta-analysis

**DOI:** 10.1038/s41598-023-27379-9

**Published:** 2023-01-11

**Authors:** Styliani Partalidou, Apostolos Mamopoulos, Despoina Dimopoulou, Theodoros Dimitroulas

**Affiliations:** 1 4th Department of Internal Medicine, Hippokration General Hospital, School of Medicine, Aristotle University of Thessaloniki, Thessaloniki, Greece; 2 Third Department of Obstetrics and Gynecology, Hippokration General Hospital, School of Medicine, Aristotle University of Thessaloniki, Thessaloniki, Greece

**Keywords:** Rheumatic diseases, Medical research, Epidemiology, Outcomes research

## Abstract

Takayasu arteritis (TA) is a systemic disease affecting women of reproductive age. Similarly to other systemic autoimmune diseases, pregnancies in patients suffering from TA are at high risk for adverse outcomes; however, the precise incidence of adverse events has not been assessed in a systematic approach. The aim of this study was to evaluate the prevalence of adverse pregnancy outcomes in TA. Searches were conducted on PubMed, Cochrane Library, Scopus and Cinahl databases from inception to 25 May 2022. Three independent investigators extracted data and assessed the risk of bias using ROBINS-1 tool. We used a random effects model to calculate the prevalence of the adverse pregnancy outcomes in TA, namely miscarriage, hypertension and pre-eclampsia. We calculated the prevalence of the adverse outcomes in pregnancy for TA. We included 27 studies, with 825 pregnancies. The occurrence of miscarriage, hypertension and pre-eclampsia in patients with TA was 16% (CI 12–21%, p < 0.01), 37% (CI 30–45%, p < 0.01) and 14% (CI 8–23%, p < 0.01), respectively. The results of our meta-analysis indicate that pregnancies in patients with TA are at increased risk for adverse pregnancy outcomes compared to the general population, suggesting that pregnant women with TA should be closely monitored.

Trial registration: There was no registration for this systematic review.

## Introduction

Systemic vasculitides are rare autoimmune disorders characterized by necrotizing inflammatory infiltration of blood vessels and multisystem involvement. Vasculitides are classified into large, medium and small vessels diseases based on the size of the involved arteries^1^. Takayasu arteritis (TA) is a large vessels vasculitis, predominantly affecting the main branches of the aorta. It is more common in women of childbearing age, approximately 20–30 years old^2,3^.

It is widely recommended that women suffering from autoimmune systemic diseases need to plan their pregnancy during periods of remission or low disease activity in order to avoid unfavorable outcomes for both mother and fetus, as well as to prevent a severe relapse of the disease itself during this period^4^. During previous decades, young women with TA were advised to avoid pregnancy, due to elevated risk of adverse events. Better understanding of the pathophysiologic mechanisms and the expansion of therapeutic choices have resulted in efficient control of the disease, including lower rates of irreversible tissue damage, better quality of life and increased life expectancy^5,6^. Despite such advances, pregnancy in such patients is still considered as high risk and requires special management and follow up^7^.

Recent evidence support that pregnant women with TA are at increased risk of hypertension, miscarriage and intrauterine growth restriction (IUGR) neonates with this excess risk to be associated with high disease activity during conception^8^. On the other hand, observations suggest that TA tends to remain stable or improve during pregnancy, once conception was achieved during period of low disease activity^9^. However, the precise effect of TA on pregnancy outcomes compared to the general population has not been assessed in a systematic, holistic approach.

The aim of this study is to synthesize the data that refer to pregnancy in women with TA, in order to provide a comprehensive meta-analysis of the impact of the disease on the pregnancy course and outcomes in this population.

## Materials and methods

This study is a systematic review and meta-analysis of proportions. The results are reported according to PRISMA guidelines^10^. We conducted a meta-analysis of proportions, as most of the studies included are observational-retrospective or prospective.

### Search strategy and inclusion criteria

A search of the open access electronic databases PubMed, Scopus, Cochrane Library, and Cinahl until 25 May 2022 was conducted. The research algorithm included the terms “Takayasu arteritis”, “systemic vasculitis” and “pregnancy outcome”, both in all fields and MeSH (medical subject headings) terms. Precisely, the search strategy was the following: ((Takayasu arteritis) OR ((systemic vasculitis[MeSH Terms]) OR (systemic vasculitis))) AND ((pregnancy outcome) OR (pregnancy outcome[MeSH Terms])).

For this systematic review and meta-analysis, we included pregnant adult women (age > 18 years old) with TA, based on either ACR or Ishikawa classification criteria^11,12^. Other group of systemic vasculitides or secondary (infective, drug-induced or malignancy-induced) were excluded. We also, excluded studies that were not published in English, case reports and papers that did not provide arithmetic data to proceed in calculations.

We included observational-prospective or retrospective and case control studies. In some, there was comparison of pregnancy outcomes before and after vasculitis diagnosis. In these cases, we used only the data after the onset of the disease, as it was considered that before disease onset, the negative results of the vasculitis would, probably, be absent.

### Data extraction

The data from the final studies were exported from two independent investigators (SP, DD) and included: first author, publication year, country, study type (prospective, retrospective, case control), number of patients and pregnancies, diagnostic criteria, mean age, mean duration of disease, medication, miscarriage (%), cesarian section (%), pre-term delivery (%), hypertension (%), pre-eclampsia (%) and IUGR neonates (%).

### Study outcomes

The outcomes studied were miscarriage, defined as loss of pregnancy before 20th week of gestation (terminations of pregnancy either for social or medical reasons, e.g., patient on therapy with possible teratogenic agents, like cyclophosphamide, were not included in this category), hypertension (> 140/90 mmHg) and pre-eclampsia^13–15^.

### Statistical analysis

A meta-analysis of proportions was conducted for every outcome, to calculate the total prevalence^16^. This meta-analysis type is used for statistical synthesis of studies that do not include control group. Although some of the studies included are case control, they were very few, so we proceeded in meta-analysis of proportions.

All calculations were conducted with RStudio (version 2022.02.3). The data were not normally distributed, so we made a logarithmic transformation. For heterogeneity evaluation we used the Ι^2^ index. When the value of this index is above 50%, it is considered that there is important heterogeneity between studies. Furthermore, for our calculations, we preferred the random effects model, which is more suitable for medical research. In cases that high heterogeneity was noticed, we found the outliers studies, according to the z-value. The limit of two was considered significant so, every study with z-value above that, was considered as an outlier that contributed much to the high heterogeneity. Studies that did not report results for a specific outcome were excluded from the synthesis^16,17^.

## Results

### Study search results

**Figure 1:**
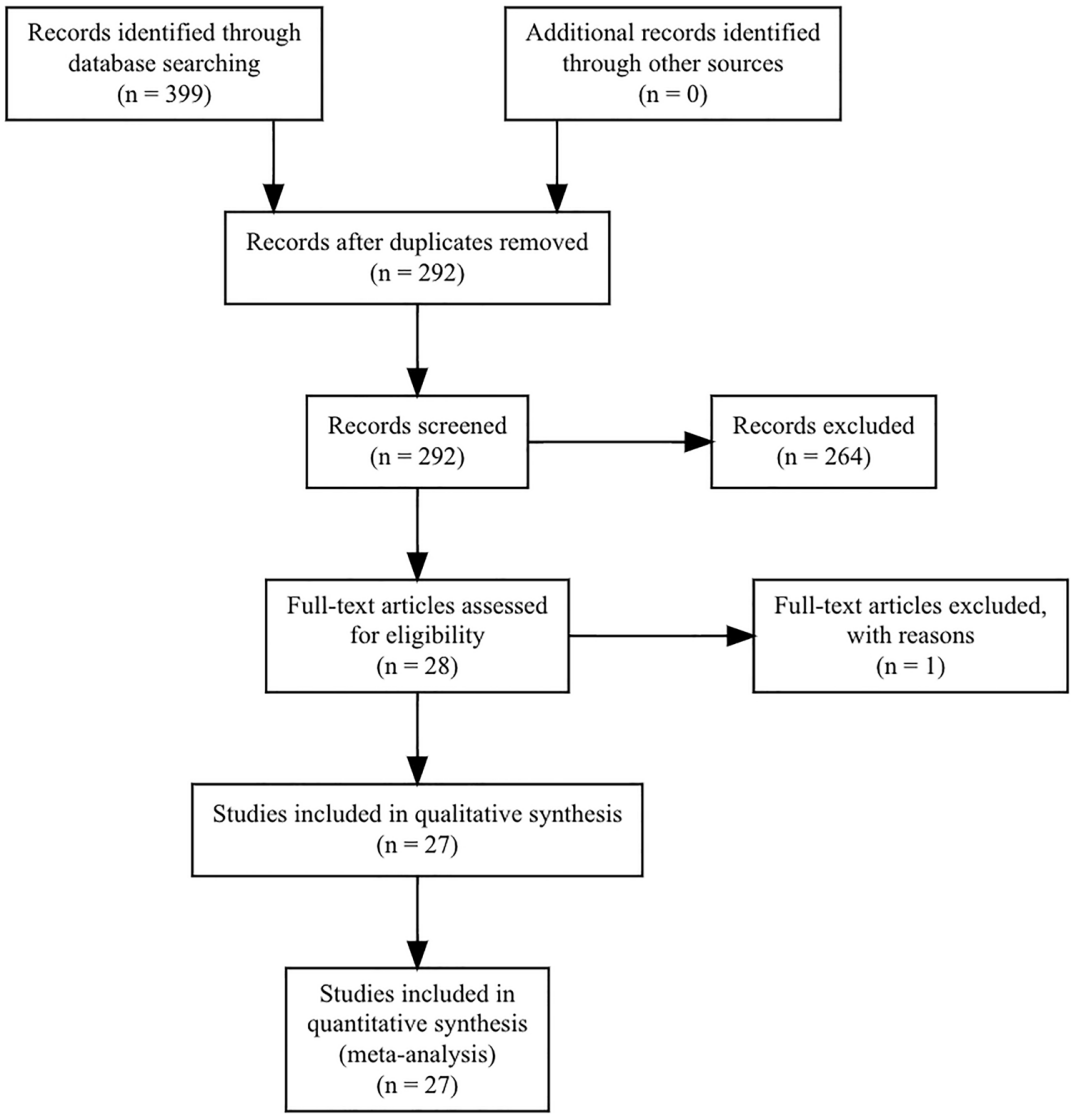
PRISMA flow diagram.

We conducted a thorough investigation of the literature and found 399 studies. After removal of the duplicates, 292 remained. Two independent investigators (SP, DD) assessed the rest at the level of title and abstract. Consensus was reached with a third investigator (AM), if any disagreement occurred, and 264 articles were excluded. The rest 28 were evaluated as a whole text. One paper was excluded, because there was no consensus on the numbers in text and in tables. We totally included 27 papers referring to TA^8,18–43^. A flow diagram according to PRISMA guidelines follows (Fig. 1).

### Study characteristics

Next, we present the basic characteristics of the studies included in the systematic review and meta-analysis (Table 1).

**Table 1:** Basic characteristics of studies included in Takayasu arteritis meta-analysis.

Study	Country	Study type	Pregnancies (Ν)	Patients (Ν)	Mean age	Medication	Miscarriage	HTN	Pre-eclampsia
Tanacan, 2018	Germany	Retrospective	22	11	24.5	CSs, LMWH, LDA	5	8	NR
Hidaka, 2011	Japan	Retrospective	26	10	29.3	CSs, LDA	5	2	NR
Ishikawa, 1982	Japan	Prospective	33	27	28	CSs	0	10	1
Wong, 1983	China	Retrospective	19	11	NR	Anti-HTN	3	10	11
Aso, 1992	Japan	Retrospective	23	15	27.5	NR	1	13	NR
Matsumura, 1992	Japan	Prospective	22	18	30.5	CSs	NR	7	NR
Sharma, 2000	India	Retrospective	24	12	23.5	Anti-HTN	5	11	4
Suri, 2010	India	Retrospective	37	15	27.6	CSs	7	10	23
Mandal, 2011	India	Retrospective	29	16	18–35	NR	2	29	21
Jesus, 2012	India	Retrospective	11	9	17–42	LDA, CSs, anti-HTN	0	7	1
Tanaka, 2014	Japan	Retrospective	27	20	30	CSs, LDA	0	6	4
Aplay-Kanitez, 2014	Turkey	Retrospective	21	36	24.5	CSs, AZA, INF	1	3	2
Singe, 2015	India	Prospective	18	12	22–28	Anti-HTN, LMWH	5	12	2
Mohamad, 2015	Sweden	Retrospective	7	10	NR	NR	1	NR	1
Assad, 2015	Brazil	Retrospective	38	NR	25.1	NR	4	12	0
Comarmond, 2015	France	Retrospective	98	52	28	LDA, CSs, anti-HTN, DMARDs	9	25	20
Zhang, 2017	China	Retrospective	13	13	30.31	NR	2	10	3
Gudbrandsson, 2016	Norway	Retrospective	37	23	29.3	NR	6	NR	1
David, 2020	India	Retrospective	NR	16	10–40	Anti-HTN, vascular surgery	NR	7	2
Shiping He, 2022	China	Case–control	110	80	27.3	LDA, CSs, AZA, anti-HTN, LMWH	36	20	2
Pyo, 2020	Korea	Retrospective	12	7	32	CSs	2	7	1
Abisror, 2020	France	Retrospective	43	33	30	CSs, AZA, INF	1	15	4
Gupta, 2020	India	Retrospective	38	20	29	NR	10	15	NR
Gönenli, 2022	Turkey	Retrospective	75	–	43.7	CSs, AZA	19	16	3
Giraldo, 2021	Colombia	Case–control	6	6	24	NR	0	5	1
Nguyen, 2019	Canada	Retrospective	12	NR	33.4	CSs, AZA, anti-HTN, surgery	1	2	2
Fredi, 2015	Italy/France	Prospective	8	6	31.7	NR	2	2	0

*HTN* hypertension, *CSs* corticosteroids, *LDA* low dose of aspirin, *LMWH* low molecular weight heparin, *anti-HTN* anti-hypertensives, *AZA* azathioprine, *IFX* infliximab, *NR* not reported.

### Risk of bias assessment

**Figure 2:**
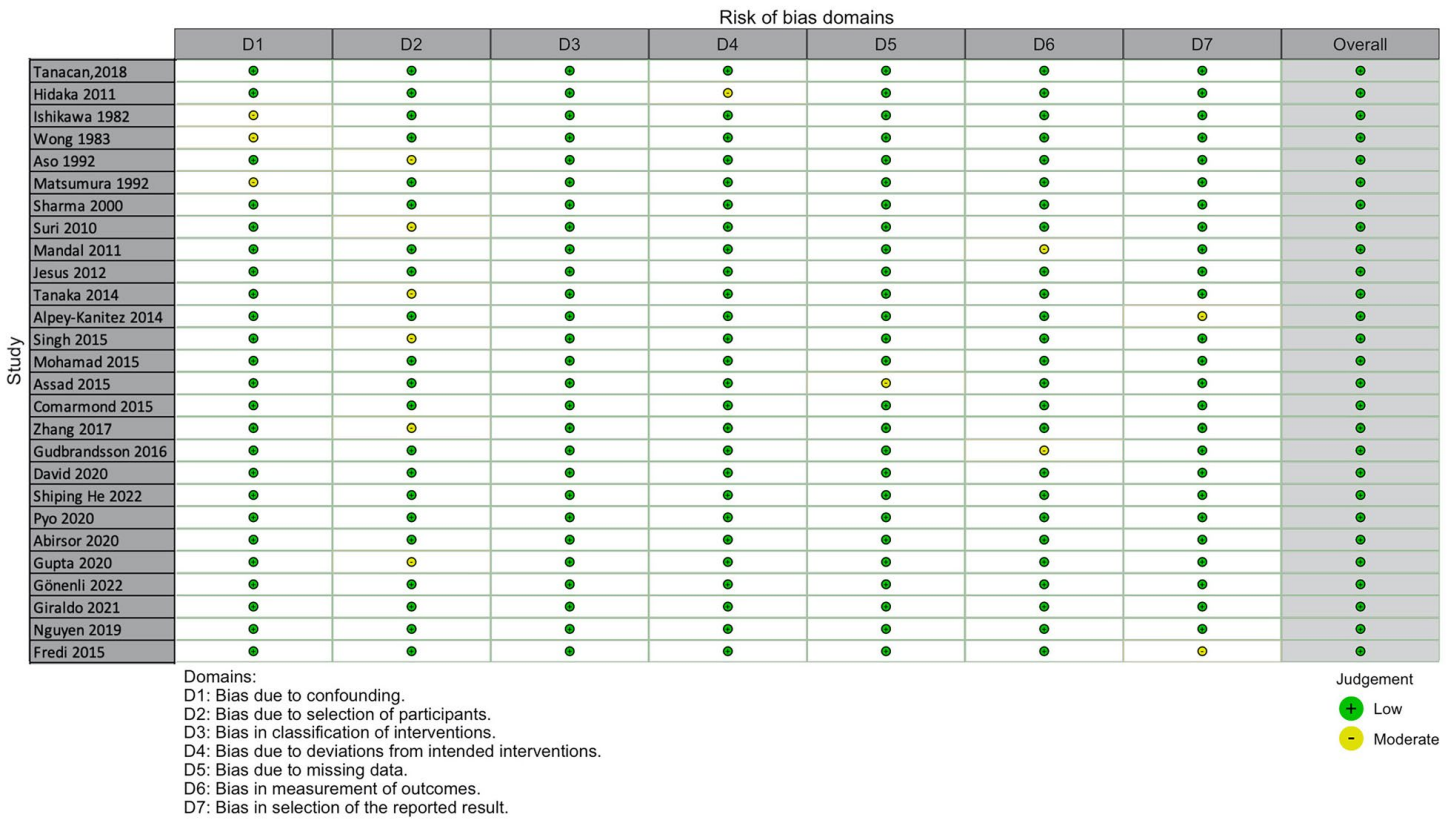
Assessment of bias for the studies included in the meta-analysis of Takayasu arteritis.

The included articles were assessed by two independent investigators (SP, AM) for risk of bias with the “ROBINS-1” tool, for non-randomized trials. This tool evaluates every study in seven domains, as presented in the figure below (Fig. 2). There was no article of low quality^44^.

### Adverse pregnancy outcomes in Takayasu arteritis

**Figure 3:**
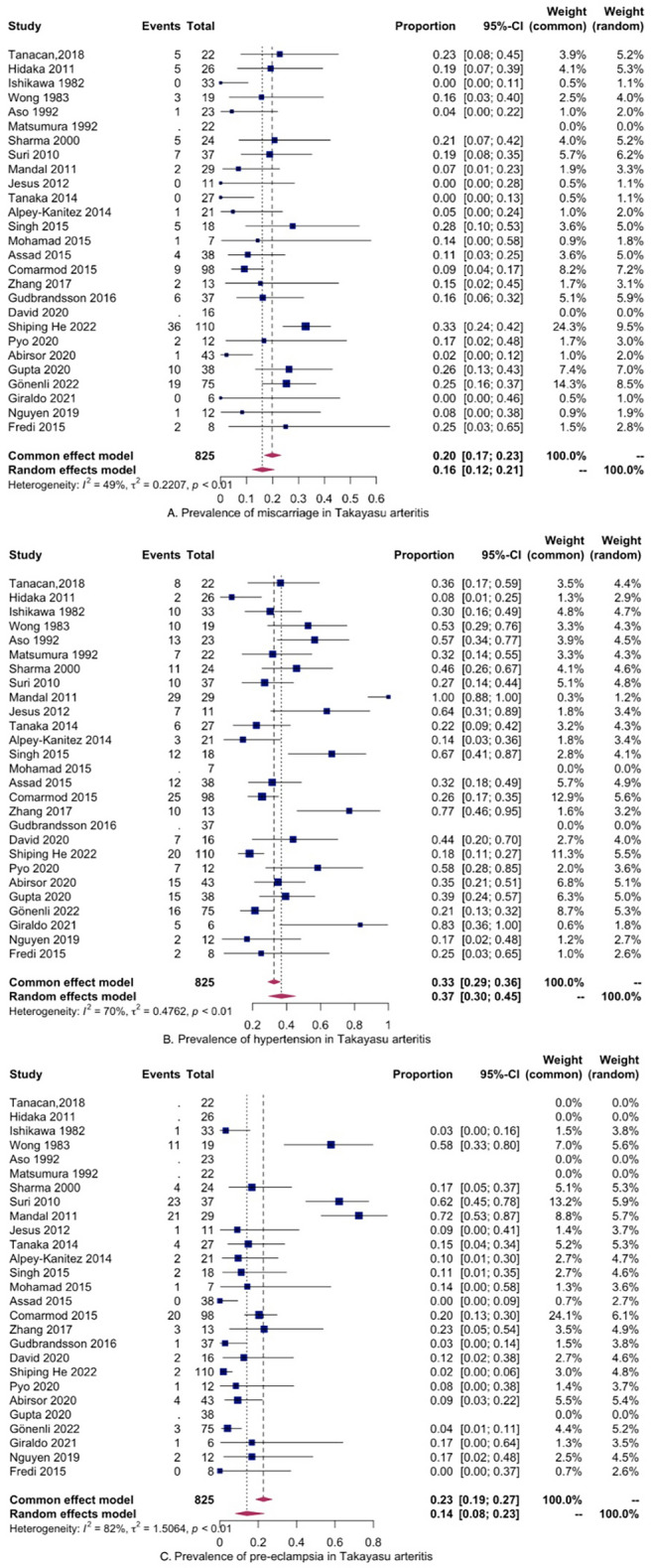
Prevalence of adverse outcomes in pregnant women with Takayasu arteritis.

Miscarriage was calculated in 16% (CI 12–21%, p < 0.01), as presented below (Fig. 3A). The two studies with missing values for this outcome (Matsumura and David) were excluded automatically^23,36^.

The prevalence of hypertension was assessed 37% (CI 30–45%, p < 0.01). Again, studies with missing data were excluded (Fig. 3B). According to z-values, we found that studies of Hidaka and Mandal contributed much to the heterogeneity^19,26^. We re-evaluated the I^2^ index after removing first Hidaka, and then Mandal study and the values were 69% and 67%, respectively. The prevalence of hypertension in TA without these two studies remained 37% (CI 30–44%, p < 0.01).

The prevalence of pre-eclampsia in TA pregnant women was calculated in 14% (CI 8–23%, p < 0.01), with relatively high heterogeneity (Fig. 3C). According to z-value, the study that diverged from the others was Mandal et al.^26^. The prevalence of pre-eclampsia after excluding this one was 12% and the I^2^ was 77.58%. Next, we present a figure with the aforementioned results.

## Discussion

The findings of the current systematic review and meta-analysis indicate a significantly higher rate of maternal complications, namely hypertension, miscarriage and pre-eclampsia occurring in 37%, 16% and 14% of pregnancies in TA women, compared to the general population. Respectively, large-scale reviews report that in community, miscarriage presents in 15%, hypertension in 5–10% and pre-eclampsia in 3%, far less than our findings^45–47^. Such observations highlight the complexity of pregnancy in TA and underscore the multidisciplinary approach in these patients.

The anchor risk factor for unfavorable pregnancy outcomes according to Shiping He et al. seems to be high disease activity during pregnancy, followed by renal artery involvement and history of hypertension^8^. On the other hand, advanced maternal age or prolonged disease duration did not predispose to obstetric complications^8^. Gönenli et al., also reported that maternal age did not oppose as risk factor for poor pregnancy outcomes, in contrast to vascular damage, and recent diagnosis, commonly associated with uncontrolled inflammation and high disease activity^40^.

New onset and/or aggravation of hypertension appear to be the main maternal complications in our analysis, which concurs with previous studies^8^. This finding was not surprising, as in the majority of patients in the studies included, hypertension was the main symptom of the disease^24,33,36^. It is worth noting that high blood pressure during pregnancy presented even in women under anti-hypertensive medication, suggesting the necessity of close monitoring despite anti-hypertensive treatment^21,24^. Of note, the incidence of pre-eclampsia is lower compared to hypertension, potentially reflecting the optimal management of pregnant women with TA. Indeed, Abisror et al., demonstrated that the administration of low dose glucocorticoids, namely 5 mg of prednisolone, resulted in lower rates of pre-eclampsia, probably via reducing the inflammatory burden^38^. In any case, the acknowledgment of the risk coupled with the efficient control of blood pressure before and during pregnancy is of utmost importance for the achievement of favorable outcomes.

With regards to low incidence of miscarriages and abortions demonstrated in our analysis, some, but not all studies have reported similar results^8,33^. Apparently, such discrepancy is related with the heterogeneity of the studies especially the disease activity status during the conception and pregnancy period.

In view of treatment, the most common prescribed regimens were corticosteroids and/or anti-hypertensive agents. Furthermore, prophylactic prescription of low dose aspirin has been decribed, although Abisror et al. did not find any beneficial effect on fetomaternal outcomes^18,27,38^ Less frequently, low molecular weight heparin was prescribed^8,20,30^. Immunosuppressive drugs, compatible with pregnancy, mainly azathioprine, have also been administrateed^29^. Finally, vascular surgery repair of affected arteries was part of the therapeutic intervention and pregnancy planning in some cases before conception^36^.

To the best of our knowledge, this is the first meta-analysis to combine data from all published studies in large databases. Given that pregnancy in TA remains a challenge for rheumatologists and obstetricians, the current findings provide in a systematic approach the most frequent complications during pregnancy in TA women. It is vital for patients to conceive during remission, or at least low disease activity, in order to decrease the risk of adverse outcomes^48^. As pregnant women with TA are predisposed to pregnancy complications, diligent surveillance especially for blood pressure, and kidney function are necessary for optimal outcome^49^.

Still, our findings should be interpreted in the context of several limitations relevant to the substantial heterogeneity among the included publications. Indeed, studies varied significantly in terms of population characteristics-especially medication schemes and disease activity, sample size and study design. Although heterogeneity was high according to the I^2^ index, we should underline that in meta-analysis of proportions, high values are expected, due to differences in place and time of study conduction. Therefore, high values of I^2^ do not, necessarily indicate inconsistent studies^16^.

Prospective case–control studies, even with small numbers, are currently lacking from recent bibliography. That prevents us from performing a classic meta-analysis that would enable us to assess in larger samples the greater risk for adverse pregnancy outcomes that these patients present, in comparison with the general population. To date, most published studies are only retrospective or small observational ones.

Our study does not only report on the incidence of pregnancy complications but, it also showcases the need for further research. Conducting high-quality observational studies, as well as exploring the impact of pregnancy in less common systemic inflammatory diseases such as vasculitides, systemic sclerosis and inflammatory myopathies is deemed mandatory to provide a realistic guidance for clinicians. It is worth noting that, in contrast to other conditions, guidelines for the management of family planning, assisted reproduction and pregnancy in TA, are currently lacking, and therefore, observational large studies focusing on potential management strategies, could provide a guidance for clinical practice^50^.

## Conclusion

Our study estimated high rates of hypertension, miscarriage, and pre-eclampsia in TA pregnant women. These findings indicate that women with TA are at higher risk of adverse pregnancy outcomes than in general population. Thus, more intense follow-up along with multidisciplinary approach is needed for these patients to reach an optimal delivery.

## Data Availability

The data supporting the conclusions of this article are in this article.

## Abbreviations

IUGR: Intrauterine growth restriction
TA: Takayasu arteritis
