# Publisher Correction: Pregnancy outcomes in Takayasu arteritis patients: a systematic review and meta-analysis

**DOI:** 10.1038/s41598-023-29324-2

**Published:** 2023-02-09

**Authors:** Styliani Partalidou, Apostolos Mamopoulos, Despoina Dimopoulou, Theodoros Dimitroulas

**Affiliations:** 1 4th Department of Internal Medicine, Hippokration General Hospital, School of Medicine, Aristotle University of Thessaloniki, Thessaloniki, Greece; 2 Third Department of Obstetrics and Gynecology, Hippokration General Hospital, School of Medicine, Aristotle University of Thessaloniki, Thessaloniki, Greece

Correction to: *Scientific Reports* 10.1038/s41598-023-27379-9, published online 11 January 2023

The original version of this Article contained errors in the Abstract.

“To evaluate the prevalence of adverse pregnancy outcomes in TA. Searches were conducted on PubMed, Cochrane Library, Scopus and Cinahl databases from inception to 25 May 2022.”

now reads:

“The aim of this study was to evaluate the prevalence of adverse pregnancy outcomes in TA. Searches were conducted on PubMed, Cochrane Library, Scopus and Cinahl databases from inception to 25 May 2022.”

Additionally,

“Trial registration: There was no registration for this systematic review. The aim of this study was to evaluate etc in order to be correct by syntax.”

now reads:

“Trial registration: There was no registration for this systematic review.”

The original Article has been corrected.

